# Excerpta

**Published:** 1880-09

**Authors:** 


### THE EVOLUTION OF CONSCIOUSNESS.

BY HORATIO R. BIGELOW, M. D., WASHINGTON, D. C.

"What weight can we attach to a man's philosophy, who, after telling us that consciousness may possibly be an inherent property of matter, of which 'the receipt of reason is a limbec only,' adds in the same breath almost, that matter generally is certainly not conscious, and that consciousness comes to the brain we know not whence nor wherefore? What shall we say of a man who in one sentence tells us that it is impossible that science can ever solve the riddle of things, and tells us in the next sentence that it is doubtful if this impossibility will be accomplished within the next fifty years? Who argues that God is a mystery, and therefore God is a fiction. Who admits that consciousness is a fact, and yet proclaims that it is a mystery; and who says that the fact of matter producing consciousness being a mystery, proves the mystery of consciousness acting in matter to be a fact?" ["Is Life Worth Living," by W. H. Mallock.] This reference to an address by Professor Tyndall, is the substance of a footnote to chapter IX, "The Logic of Scientific Negation," of Mr. Mallock's masterly thesis. I could wish for nothing better in the great struggle of enlightenment than that this book might be in the hands of every one interested in the drift of modern positivism. It is well conceived and admirably carried out. Its logic is keen, its sentences terse and clearly cut, its deductions unimpeachable. To physiologists it is of especial interest, because upon the results of cultured physiological inquiry will rest the ultimate rise or fall of positivist theory.

It is not within the scope of the present article to discuss the question of Truth and Happiness—whether the possession of the former conduces to the latter, or whether the attainment of the latter will necessarily rest upon the former—though he who ministers to a mind diseased must be intimate with the various phenomena which go to make up the action of the mind in health. It is apparent, however, that these terms have no permanent value, since their definition must vary with each one's interpretation, and since their demonstration in fact is not possible. The mystery of the supernatural enwraps them, just as it enwraps the mystery of life itself. There is a point of "inchoate consciousness" at which all scientists have confessed themselves arrested, and it is just here where the modern school of skepticism will ask us to accept an idealistic theory in lieu of the faith which they decry in matters of theological polemics.

Never was human parturition so fraught with agony as is the labor of a positivism—limited because finite—striving to give birth to an intellectual conception of the incomprehensible, which, being infinite, in the sense of not being material, may never be measured by finity. Just at the point where matter ends and mind begins we are asked to accept a scientific creed, belief in which will entail a larger amount of faith than Christianity demands of its adherents. If consciousness be evolved from a surrounding conscious nature, to which it shall return, we should expect to find in that nature a harmonious perfection—the consummation of truth. No one, as yet, has ventured to offer so untenable an explication. Nature, in her inconsistencies, horrifies us at every turn. Is consciousness, then, the result of molecular change in the brain, or does the presence of consciousness cause the molecular change? It may not be questioned that the brain is the organ through which consciousness is made manifest; and this admission is entirely consistent with theistic doctrine. But if it be argued that the outcome of cerebral molecular change is consciousness, then should the physical processes be complete in themselves, and go on and on with the same integrity as now, even were consciousness absent. Dr. Tyndall says: "Do states of consciousness enter as links into the chain of antecedence and sequence which gives rise to bodily actions? * * * I have no power of imagining such states interposed between the molecules of the brain, and influencing the transferrence of motion among the molecules. But the production of consciousness by molecular action is quite as unpresentable to the mental vision as the production of molecular motion by consciousness. If I reject one result I reject both. I, however, reject neither and thus stand in the presence of two incomprehensibles, instead of one incomprehensible." (Quoted by Mallock.)

The only possible and rational explanation of the existence of consciousness is the belief that the brain is the natural organ, attuned by supernatural fingers; and since inherent potentiality in matter is not demonstrable, and molecular motion cannot originate of itself, and never has so originated, it requires a less degree of faith to believe that "such states are interposed between the molecules of the brain, influencing the transferrence of motion among the molecules,' than to believe that the production of consciousness is due to an inherent, indestructible property of the molecule to originate that motion which we call consciousness. The existence of the material and of the immaterial or spiritual are unconsciously admitted by Dr. Tyndall. The first incomprehensible, of molecular motion, is material; the second incomprehensible, of consciousness originating molecular change, is immaterial. Neither result does Dr. Tyndall reject, hence he must tacitly accept "interpretation of grosser minds," that "heathen notion," of a soul. At this threshold, where through the gate that is half ajar there streams an effulgent ray from the Infinite Himself, the Professor views his own insufficiency and professes that he does not reject the theory of the supernatural. The fashioning of our own consciousness pre-supposes an infinite power of creation out of a

Supreme Consciousness, to which our immortality is allied. This potentiality has not been given to man, and a self conscious molecule is a scientific vagary unworthy consideration. " Every thought and feeling has its definite mechanical correlative, that is accompanied by a certain breaking-up and re-marshaling of the atoms of the brain." [Dr. Tyndall.] This may be granted without reservation, but can Dr. Tyndall tell us whether the chemico-molecular action precedes thought, or whether a power which is as yet to us a mystery induces the molecular change, the outcome of which is thought? Can the microscopist define its characteristics? Can the physiologist tell us just what re-actions of molecules are necessary to perfect certain trains of thought? Will any positivist trace out the cerebral conditions, together with the chemical formulæ, engendering that condition which will lead us to the attainment of his consummation or morality —happiness?

In every department of exact inquiry there is an unconscious appeal to the supernatural, and positivists are constantly making use of terms which are absolutely devoid of meaning, unless a belief in the supernatural be implied. No man can explain the nature, cause and end of consciousness. Its unprejudiced contemplation leads up higher and higher, until in the presence of its Infinite Creator we find that peace and consolation which is the comforting assurance of an immortality in which we shall know as we are known.

In this hope is the Christian's creed. Theirs is no impossible ideal, no theoretic fancy, that truth for truth's sake is heroic, and that the end of human li e is a happiness, which, be it said, no positivist confesses as yet to have attained. Their Truth finds its ultimate consummation in the contemplation of the Infinite Truth, and their happiness lies in immortality.

But to admit that in consciousness we have a proof of a spiritual governance, were to adjure the whole positivist creed; and hence we are asked to accept as explanatory a premise in every way most unscientific.

Until it may be demonstrated that molecules may so interact as to produce consciousness, it is nobler, as it is more logical, to confess our inability to trace its cause, and to believe, what may not be disproved, that a Higher Power governs it, and that by its presence molecular change is engendered.—*Philadelphia Medical and Surgical Reporter.*

FEMALE PHYSICIANS IN THE OLDEN TIME.—After all it is nothing new to have female physicians. Even the earliest records of the world's history bear testimony to instances of the successful practice of medicine by women. Mythology corroborates woman's capacity for this career by ascribing to the Egyptian Isis the duty of watching over the human species and the discovery of beneficial drugs. Among the Romans, Juno Lucina presided over childbirth and hastened delivery. Hygeia, the daughter of Esculapius, and Ocyroe, the daughter of Chiron, were learned in medicine. Esculapius portrayed as followed by a multitude of both sexes who dispensed his benefits. As early as the eleventh century before Christ there existed in Egypt a college of physicians, who seem to have been of the sacerdotal caste, and was attended by both sexes. The "Iliad" and "Odessey" both refer to women skilled in the science of medicine; among the Greeks, Olympias, of Thebes, Aspasia, and Agnodice were pre-eminent for their ability and medical writings. The skill of Agnodice is said to have been such as to have brought about the legal opening of the medical profession to all free-born women of the state. Thænarete, the mother of Socrates, was a midwife. Between the eleventh and thirteenth centuries, several women acquired widespread renown as teachers in the great school of Salerno. In the succeeding centuries many female physicians held professional chairs in the universities of Italy, especially that of Bologna. In this university, about the middle of the eighteenth century, "there was an Ann Morandia Mazzolini, whose husband held the chair of anatomy. It happened that he fell ill, and she, being a loving wife, sought to supply to him the place of his enfeebled powers. So she became an anatomist, and presently delivered his lectures for him from behind a curtain. She became famous, and was offered a chair at Milan, which, however, she refused, and remained at Bologna till her death in 1774. Her anatomical models in wax are the pride of the Anatomical Museum at Bologna."—*Practical American.*

DEATH OF GEORGE WASHINGTON.—The certificate of Drs. Craik and Dick, the physicians who attended George Washington at the time of his death, has just been un-earthed, from a Georgetown newspaper of 1799. It does not appear in any of the biographies of Washington. The certificate concludes thus: "He was fully impressed at the beginning of his complaint, as well as through every succeeding stage of it, that its conclusion would be mortal; submitting to the several exertions made for his recovery rather as a duty than from any expectations of their efficacy. He considered the operations of death upon his system as coeval with the disease; and several hours before his decease, after repeated efforts to be understood, succeeded in expressing a desire that he might be permitted to die without interruption. During the short period of his illness he economized his time in the arrangement of such few concerns as required his attention with the utmost serenity, and anticipated his approaching dissolution with every demonstration of that equanimity for which his whole life has been so uniformly and singularly conspicuous."

THE TANNER FAST finally became very absurd. For about fourteen days Tanner is represented to have taken no water. Being unable to endure such an ordeal, he then commenced taking about 48 ounces per diem. In four days he imbibed say about 192 ounces. During this period he lost by the kidneys 100 ounces, and by the skin and lungs about the same amount; that is in four days he ingested a little less than 200 ounces, and he lost by the kidneys, skin and lungs, a little more than this amount; and yet in these four days he gained four pounds. As this gain can not be attributed to the fluid swallowed, to what source is it to be credited?

It is strange that while this pitiable individual was voluntarily endangering his life both immediately and prospectively, while this manifest fraud of a fast was being enacted, and this contemptible travesty upon science was being displayed, there were persons supervising it who call themselves physicians! If these men could render themselves thus absurd, could they not have sufficient generosity to do this without raising over themselves the banner of science?

PREVENTION OF LACERATED PERINEUM.—B. E. Mossman advocates artificial dilatation of the perineal structures before the head reaches the floor of the pelvis, in order to prevent laceration. He claims that his method has never failed, in uncomplicated labor in normal primiparæ to prevent rending so much as even the mucus membrane covering the inner sides of the fourchette.

He annoints the external parts and the vagina as far as the finger will go, with melted lard with extracts of belladonna; and if the first stage of labor occupies one or two hours, he makes two or three such applications. As soon as the womb has dilated sufficiently so that the crevix is safe against laceration, he begins at once artificial dilatation of the perineum. He applies the belladonna ointment freely, and then places one or two fingers within the vagina, making pressure lightly but continuously downward and forward.

When the head descends so as to press upon the perineum, he removes the finger from the vagina, and introducing them into the the perineum forward and upward, and presses the head upward under the pubes whenever a pain comes on, Goodell's method of protecting the perineum.

When the pain ceases and the head recedes, he applies the dilating force with the fingers in the vagina as before, alternating the pressure from within with the forward traction during the pain, and retarding the expulsion of the head until the dilatation is sufficient to allow the escape of the head without laceration.

He thinks that it is very rare that shoulders cause laceration after the head has safely passed.—*American Jour. of Obstetrics.*

Treatment of Anal Fissure.—Dr. Hamon informs us, in *Le Practicien*, that instead of employing forcible dilatation, he applies to the fissure, with a camel-hair brush, a solution consisting of one part of chloroform to two parts of alcohol. Two or three applications, at intervals of two or three days, usually suffice to effect a cure. The first application is very painful, but each subsequent one becomes less so.—*Southern Practitioner.*

Bromide of Potassium.—How many patients refuse to continue the use of Bromide of Potassium from its intensely bitter salt taste? This taste is easily overcome by giving ℥ iii of simple syrup with each drachm of the bromide. The ℥ iii of syrup, if properly made, should contain about 150 grains of sugar. This completely alters the taste, giving it an agreeable nutty flavor, not unlike to cocoanut milk, if largely diluted. Children take it with avidity, The sugar in no way alters the medicinal virtues of the drug. This is a boon to epileptics and others who have to persevere in large doses of the bromide.—*Canada Medical and Surgical Journal.*

### JULY REPORT OF DEATHS AND INTERMENTS IN BALTIMORE.

| CAUSE OF DEATH. | No. | CAUSE OF DEATH. | No. | CAUSE OF DEATH. | No. |
|---|---|---|---|---|---|
| Abscess | 2 | Drowned | 12 | Marasmus | 38 |
| " Cervical Gland, | 1 | Dysentery | 12 | Malnutrition | 6 |
| " Liver | 1 | Excessive use of Opium | 1 | Measles | 6 |
| Anæmia | 1 | Exhaustion | 1 | Malarial Cachexis | 1 |
| Apoplexy | 17 | Erysipelas | 2 | Meningitis | 14 |
| Asphyxia | 1 | Entero Colitis | 6 | " Tubercular | 6 |
| Asthma | 3 | Exposure | 1 | " Cerebro Spinal | 4 |
| Asthenia | 11 | Endo Garditis | 2 | Murder | 1 |
| Bright's Disease | 12 | Fever, Brain | 1 | Nervous Prostration | 1 |
| Burns | 1 | " Intermittent | 2 | Old Age | 29 |
| Capillary Bronchitis | 1 | " Malarial | 4 | Overcome by Heat, | 1 |
| Carbuncle | 1 | " Puerperal | 1 | Paralysis | 10 |
| Cancer, Diffused | 1 | " Remittent | 3 | Pneumonia | 15 |
| " Face | 1 | " Sca let | 36 | " Typhoid | 1 |
| " Breast | 1 | " Typhoid | 26 | " broncho | 1 |
| " Axille | 1 | " Typho-Malarial | 3 | Prolonged Labor | 1 |
| " Omentum | 1 | Fall from Rigging | 1 | Premature Birth | 14 |
| " Uterus | 5 | Fracture Thigh | 1 | Progress Locomotis Ataxia. | 1 |
| " Stomach | 5 | " Skull | 2 | Purpura | 1 |
| Catarrh of Bladder | 1 | " Vertebræ | 1 | Puerpural Metritis | 1 |
| Child Birth | 2 | Gangrene Lungs | 1 | " Peritonitis | 1 |
| Cholera Infantum | 169 | " Senile | 1 | Rheumatism of Heart | 1 |
| " Morbus | 9 | Gastro Enteritis | 11 | Scrofula | 6 |
| Cirrhosis of Liver | 2 | Goitre | 1 | Syncope | 1 |
| Concussion of Brain | 1 | Hernia Strangulated | 1 | Softening of Brain | 7 |
| Congestion of Brain | 10 | Hemorrhage Lungs | 1 | Stricture of Intestines | 1 |
| " Internal | 1 | " Uterus | 1 | Scalds | 1 |
| " Lungs | 1 | " Umbilical | 1 | Sun Stroke | 6 |
| Consumption of Bowels | 1 | Hydrocephalus | 8 | Suicide | 3 |
| Consumption of Lungs | 118 | Inanition | 28 | Syphilis | 2 |
| Convulsions | 35 | Insanity | 1 | Tabes Mesenterica | 3 |
| ,, Puerperal | 2 | Inflamation of Brain | 1 | Thrust | 2 |
| Congestive Chill | 1 | " Bladder | 2 | Tetanus | 2 |
| Croup | 6 | " Bowels | 10 | Traumatic Peritonitis | 1 |
| Cyanosis | 4 | " Stomach | 5 | Trismus Nascentium | 4 |
| Dentition | 27 | " Bronchi | 3 | Tumor Ovarian | 1 |
| Delirium Tremens | 2 | " Kidneys | 1 | Ulcer of Rectum | 1 |
| Diarrhœa | 19 | " Liver | 3 | Uræmia | 4 |
| Diphtheria | 20 | " Pericardium | 1 | Unknown Adult | 3 |
| Disease of Brain | 2 | " Pieura | 1 | " Infantile | 3 |
| " Heart | 17 | " Ovary | 1 | Whooping Cough | 17 |
| " Kidney | 1 | Intussusception | 1 | Wounds | 1 |
| " Liver | 1 | Intemperance | 2 | Effusion of Brain | 1 |
| " Prostrate |  | Jaundice | 1 |  |  |
| " Stomach | 1 | Leucocythermia | 1 |  | 692 |
| " Spine | 1 | Malformation | 2 |  |  |
| Dropsy, (Gen'l) | 2 | Laryngeal Phthisis | 1 |  |  |

### THE DEATHS REGISTERED IN THE MONTH INCLUDE.

| | | | |
|---|---|---|---|
| Under 1 year | 356 | Between 20 and 30 " | 61 |
| Between 1 and 2 years | 104 | " 30 and 40 " | 68 |
| " 2 and 5 years | 50 | " 40 and 50 " | 66 |
|  |  | " 50 and 60 " | 50 |
| Under 5 years | 510 | " 60 and 70 " | 54 |
| Between 5 and 10 years | 43 | " 70 and 80 " | 35 |
| " 10 and 15 " | 19 | " 80 and 90 " | 23 |
| " 15 and 20 " | 30 | " 90 and 100 " | 3 |

### NATIVITY, ETC.

| | |
|---|---|
| United States, White | 611 |
| Colored | 222 |
| Foreign | 129 |
|  | 962 |
| Still Birth | 60 |

Among the decedents were 67 above the age of 70 years, whose combined ages were 5,332 years, averaging 79 years, 7 months.

Estimated Population, 393,796. White, 338,384. Colored, 55,412. Annual Death Rate during the month, whole population, per 1,000—29.04. White, 2'1,69. Colored, 48.43.

By Order: A. R. CARTER, Secretary.

JAMES A. STEUART, M. D., Commissioner of Health and Registrar.

U. S. SIGNAL OFFICE, BALTIMORE, MD., August 1, 1880.

### METEOROLOGICAL SUMMARY FOR THE MONTH OF JULY, 1880.

#### PRESSURE.

Mean Monthly Barometer - - - - - 30.005 inches.
" " " - - - for past 10 years 29,997 inches.
Highest Barometer - - - - - - - 30,211 inches, on the 31st.
Lowest " - - - - - - - - 29,675 inches, on the 16th.
Monthly Range of Barometer - - - - - 0,536 inches.

#### TEMPERATURE.

Mean Monthly Thermometer - - - - 77.6 degrees.
" " " - - for past 10 years 78.7 degrees.
Highest Thermometer - - 99 degrees on 13th. Monthly Range - - 37 degrees.
Lowest " - - - 62 " " 30th. Mean Daily Range - 16.7 "

#### WIND, ETC.

Prevailing Direction of Wind, Northwest.
Mean Velocity of Wind, 5.3 miles per hour.
Highest Velocity of Wind, 22 " "

Total Amount of Rainfall, 6.47 inches.
Number of Days on which Rain fell, 13.
Mean relative cumidity - 64.1 per cent,

ROBERT SEYBOTH, in charge.

